# Theory of planned behavior constructs are associated with willingness to engage in clinical trial interventions for chronic low back pain: A cross-sectional survey study

**DOI:** 10.1017/cts.2025.10209

**Published:** 2025-12-19

**Authors:** Caleb Steeby, Caroline S. Zubieta, Sana Shaikh, Guohao Zhu, Jennifer Pierce

**Affiliations:** Department of Anesthesiology, University of Michigan, Ann Arbor, MI, USA

**Keywords:** Theory of planned behavior, clinical trials, chronic low back pain, retention, recruitment

## Abstract

**Background/Objective:**

Chronic pain research studies are important for both finding new treatments and improving existing treatments for individuals with chronic pain. For clinical trials to be effective, participants need to be engaged and willing to participate in treatment groups. Our research applies the theory of planned behavior (TPB) to understand how attitudes, perceived social norms, and perceived control over intervention engagement are associated with willingness to participate in interventions for chronic low back pain (CLBP).

**Methods:**

Adult Michigan Medicine patients were identified using electronic medical records and emailed a link to an online, cross-sectional survey. Participants who self-reported CLBP, ability to read and write in English, and consented to participate were able to complete the survey (*N* = 405).

**Results:**

The results showed more positive attitudes, positive social norms, and higher perceived behavioral control related to specific chronic low back pain interventions are associated with greater willingness to participate after controlling for demographic and pain-related characteristics.

**Conclusion:**

The findings suggest that TPB constructs may be useful in guiding recruitment efforts for chronic pain intervention trials.

## Introduction

Chronic low back pain (CLBP) is the leading cause of disability worldwide, affecting a total of 7.4% of the population or approximately 540 million people globally [1]. Clinical trials are essential for understanding and optimizing treatments for CLBP. However, recruitment for clinical trials is often slow [2], retention is often poor [3], many studies fail recruitment goals [3–4], and many participants may not want to participate in particular treatment arms [5]. Understanding potentially modifiable factors that impact willingness to participate in clinical trials for chronic pain may improve recruitment and retention strategies.

Demographic characteristics such as age, gender, race, and socioeconomic status [6–7], as well as pain-related characteristics, may be associated with willingness to participate in clinical trials. Previous research further suggests that levels of trust, knowledge of research, and co-occurring physical and mental health conditions may be associated with willingness to participate in clinical trials [6–10]. However, limited research has explored these associations in the context of chronic pain trials.

The theory of planned behavior (TPB) may be a useful extension to understand willingness to participate in clinical trials [11]. The TPB has been utilized extensively to understand engagement in health promotive behavior [12]. The theory suggests that intentions to engage in a particular behavior are a function of positive attitudes toward the behavior, positive perception of social norms related to the behavior, and perceived control over the behavior. To our knowledge, the TPB has not been explicitly applied to willingness to participate in clinical trials for CLBP. However, previous research suggests that its components are important [13–14]. Indeed, positive attitudes (e.g., perceived benefits of participation) and high control (e.g., low burden) are conceptualized as important facilitators for research participation [15]. For example, in a study looking at willingness to participate in breast cancer trials, women who reported a greater impact of positive aspects of research participation and less impact of negative aspects of participation were more willing to participate [13]. Gouveia et al. also found that individuals who agreed to participate in a clinical trial reported greater perceived self-efficacy compared to those who declined participation [14]. Furthermore, previous research related to personal engagement in pain interventions suggest that positive attitudes, perceived norms, and perceived control are associated with willingness to utilize treatment [16–19], however, this was not within the context of clinical trial participation.

Applying the TPB to better understand willingness to participate in clinical trials may be a useful step in identifying modifiable factors to improve recruitment and retention methods. The present cross-sectional survey was conducted as a quality improvement project to improve recruitment efforts for a CLBP clinical trial testing four interventions: mindfulness-based stress reduction (MBSR); acupressure; physical therapy; and duloxetine. We hypothesized that TPB constructs, namely positive attitudes toward the intervention, perceived positive social norms related to the intervention, and perceived control over completing the clinical trial would be associated with greater willingness to participate in the clinical trial beyond the influence of demographic and pain-related characteristics.

## Method

### Participants and procedure

This cross-sectional survey study was conducted as a quality improvement and staff training and engagement project [20] to improve recruitment efforts for an ongoing intervention study. This survey included questions regarding the interventions of the ongoing intervention study; however, the present study was a separate cross-sectional survey study and was not connected to the intervention study. Potential participants for the current study were identified using a self-service data tool that interfaces with electronic medical records at Michigan Medicine (i.e., DataDirect). Individuals over the age of 18 and who had visited either Family Medicine or the Back & Pain Center at Michigan Medicine within the past 5 years (since January 2018) were emailed a unique link to an online survey. The email stated this was a one-time online survey to better understand thoughts and attitudes about medical research related to pain treatment. After clicking on the survey link, inclusion and exclusion criteria were first presented. Individuals were asked if they could read English; if not, they were excluded. They were also asked if they had CLBP defined as any persistent or recurrent low back pain present for the last 3 months or longer; if yes, they could proceed. Those who met the criteria then completed informed consent, in the form of a yes or no question, prior to accessing the survey. Individuals who could not read English, did not have CLBP, or who did not provide consent were redirected to the survey end. Participants were able to close the survey at any point if they did not want to answer a question. The study was approved by the Michigan Medicine Institutional Review Board (HUM00228846).

## Measures

### Demographic characteristics

Participants self-reported age, gender, race, and income. Income was dichotomized to include 0 (less than 75,000 USD) and 1 (75,000 USD or more).

### Past research participation

Past research participation was assessed with a single question: *Prior to taking this survey, about how many previous research studies have you participated in?* Response options ranged from 0 (None) to 13 (25+). Response options 0 to 9 represented the corresponding number of studies. Response option 10 indicated 10–15; 11 indicated 15–20; 12 indicated 20–25; and 13 indicated 25+. The mean for past research participation was calculated as an average on the scale from 0 to 13.

### Physical function

Physical function was assessed with the 4-item PROMIS Physical Function – Short-Form 4a [21]. Participants were asked to reflect on their physical function in the past 7 days. A sample item includes: *Are you able to do chores such as vacuuming and yard work?* Response options ranged from 1 (Unable to do) to 5 (Without any difficulty). Items were summed for a total score ranging from 4 to 20. Higher scores indicate better physical function.

### Anxiety and depressive symptoms

Anxiety and depressive symptoms were assessed with the PROMIS Anxiety – Short-Form 4a and PROMIS Depression – Short-Form 4a, respectively [22]. Participants were asked to consider symptoms that have been present for the past 7 days. Sample items include: *I felt fearful* (anxiety symptoms) and *I felt worthless* (depressive symptoms). Response options ranged from 1 (Never) to 5 (Always). Sum scores were obtained ranging from 4 to 20. Higher scores indicate higher anxiety or depressive symptoms.

### Pain intensity

Pain intensity was assessed with the single item PROMIS Numeric Rating Scale v1.0 – Pain Intensity – 1a [23]. The item asks participants to consider their pain in the past 7 days. The item stated: *How would you rate your pain on average?* Response options ranged from 0 (No pain) to 10 (Worst pain imaginable).

### Intervention knowledge

For each intervention, participants were asked: *How would you rate your knowledge about [intervention] as a therapy for pain?* The interventions included MBSR, acupressure, physical therapy, and duloxetine. Response options ranged from 1 (Very low) to 5 (Very high).

### TPB constructs

The TPB specifies that its related constructs must be specific to the behavior in question [24]. Thus, TPB constructs are evaluated using unique, behavior-specific questions rather than any established measure [24]. Items to assess positive attitudes, perceived positive social norms, and perceived control over participating in each particular intervention were developed specifically for this study and are contextualized regarding target and action (research participation and required activities) and time (amount of time required to invest) for each hypothetical intervention. For each set of questions, participants were presented with additional information about the intervention and what study tasks would be required (see Supplemental Table 1). The unique intervention was inserted into each question set below (MBSR, acupressure, physical therapy, and duloxetine).

#### Positive attitudes

Positive attitudes were assessed with three items, each with the same stem: *My participation in a research study where I receive [INTERVENTION] for my low back pain would be…* Response options included: 0 (Bad for my pain) and 1 (Good for my pain); 0 (Bad for advancing science) and 1 (Good for advancing science); and 0 (Unpleasant for me) and 1 (Pleasant for me). Items were summed for a score ranging from 0 to 3.

#### Positive social norms

Positive social norms were assessed with three items: *My doctor would approve of me doing a research study that involved [INTERVENTION]; My family and friends would approve of me doing a research study that involved [INTERVENTION];* and *Most people like me would do a research study that involved [INTERVENTION].* Response options for each question were 0 (Disagree) and 1 (Agree). Items were summed for a score ranging from 0 to 3.

#### Perceived control

Perceived control was assessed with two questions: *Considering what would be required if you participated in a study with [INTERVENTION], how easy would it be to complete all of the tasks required of you?;* and *Considering what would be required if you participated in a study with [INTERVENTION], how easy would it be to overcome any obstacles to completing the tasks (e.g., lack of time, pain and fatigue that may prevent participation, etc.)?* For each intervention, specific intervention related tasks were specified. For MBSR, it stated: *8 weekly 1-hour sessions, 1 weekend retreat, and up to one hour of daily homework*. For acupressure, it stated: *30 minutes of self-administered acupressure each day in addition to 3–5 min to record in a daily log*. For physical therapy, it stated: *ten one-hour sessions: 2 sessions/week for the first 2 weeks and 1 session per week for the following 6 weeks*. For duloxetine, it stated: *taking up to two pills daily at similar approximate times each day for about 8 weeks.* Response options for each question included 0 (Difficult) and 1 (Easy). Responses were summed for a score ranging from 0 to 2.

### Willingness to participate

For each intervention (MBSR, acupressure, physical therapy, and duloxetine), participants were asked: *Now, think about participating in a research study that included the 4 interventions (Mindfulness-Based Stress Reduction, Acupressure, Physical Therapy, or Duloxetine). If it was 100% certain that you would be asked to use [INTERVENTION], how willing would you be to participate?* Response options ranged from 0 (Not at all) to 4 (Definitely).

### Data analysis plan

Descriptive statistics were evaluated for all predictors and outcomes of interest. A series of ordinal logistic regression analyses were conducted, including both univariable and multivariable models, to assess associations between predictor variables and the ordinal outcome. The results from the multivariable models served as the primary basis for interpretation, as they adjust for potential confounders. To address the issue of multiple comparisons, the False Discovery Rate (FDR) correction was applied to the p-values obtained from the multivariable analyses, ensuring robustness against type I errors. Responses were required for all scales after the demographic items; therefore, missingness would only occur when an individual exited the survey. Analyses were conducted using R 4.4.0. Data, analysis code, and output are available on Open Science Framework for transparency https://osf.io/7u5dr/view_only=de156ca102d0414a89a41270410453f6.

## Results

Four hundred five participants completed the survey (M_age_ = 52.45, SD = 16.87; 72% female; 47% White). Descriptive statistics are provided in Table 1. On average, participants reported high physical function, low anxiety and depressive symptoms, and moderate pain severity. Knowledge of each intervention was low or moderate, except for physical therapy which was generally high.

**Table 1.** Descriptive statistics for study sample

		Non-missing *n*	% (*n*)	M (SD)	Range
Age		405		52.45 (16.87)	19:89
Gender		404			
	Male		25 (101)		
	Female		72 (292)		
	Transgender/non-binary		2.7 (11)		
Race		401			
	White		47 (189)		
	Asian		12 (50)		
	Black		26 (104)		
	Other		14 (58)		
Income over 75,000 USD		397	47 (185)		
Past research participation^a^		401		1.55 (2.19)	0:13
Physical function^b^		395		13.78 (4.36)	4:20
Anxiety symptoms^b^		390		8.26 (3.83)	4:20
Depressive symptoms^b^		390		7.44 (3.94)	4:20
Pain intensity^b^		385		5.40 (2.18)	0:10
Knowledge of MBSR		369			
	Very low		33 (121)		
	Below average		24 (90)		
	Average		25 (94)		
	Above average		13 (47)		
	Very high		5 (17)		
Knowledge of acupressure		356			
	Very low		22 (78)		
	Below average		26 (91)		
	Average		38 (135)		
	Above average		12 (41)		
	Very high		3 (11)		
Knowledge of physical therapy		352			
	Very low		2 (6)		
	Below average		2 (8)		
	Average		30 (105)		
	Above average		40 (141)		
	Very high		26 (92)		
Knowledge of duloxetine		348			
	Very low		50 (173)		
	Below average		16 (54)		
	Average		18 (61)		
	Above average		10 (36)		
	Very high		7 (24)		

^a^Mean represents the average on a scale of 0 to 13.

^b^Higher scores indicate higher levels of physical function, anxiety and depressive symptoms, and pain severity.

**Figure 1.**
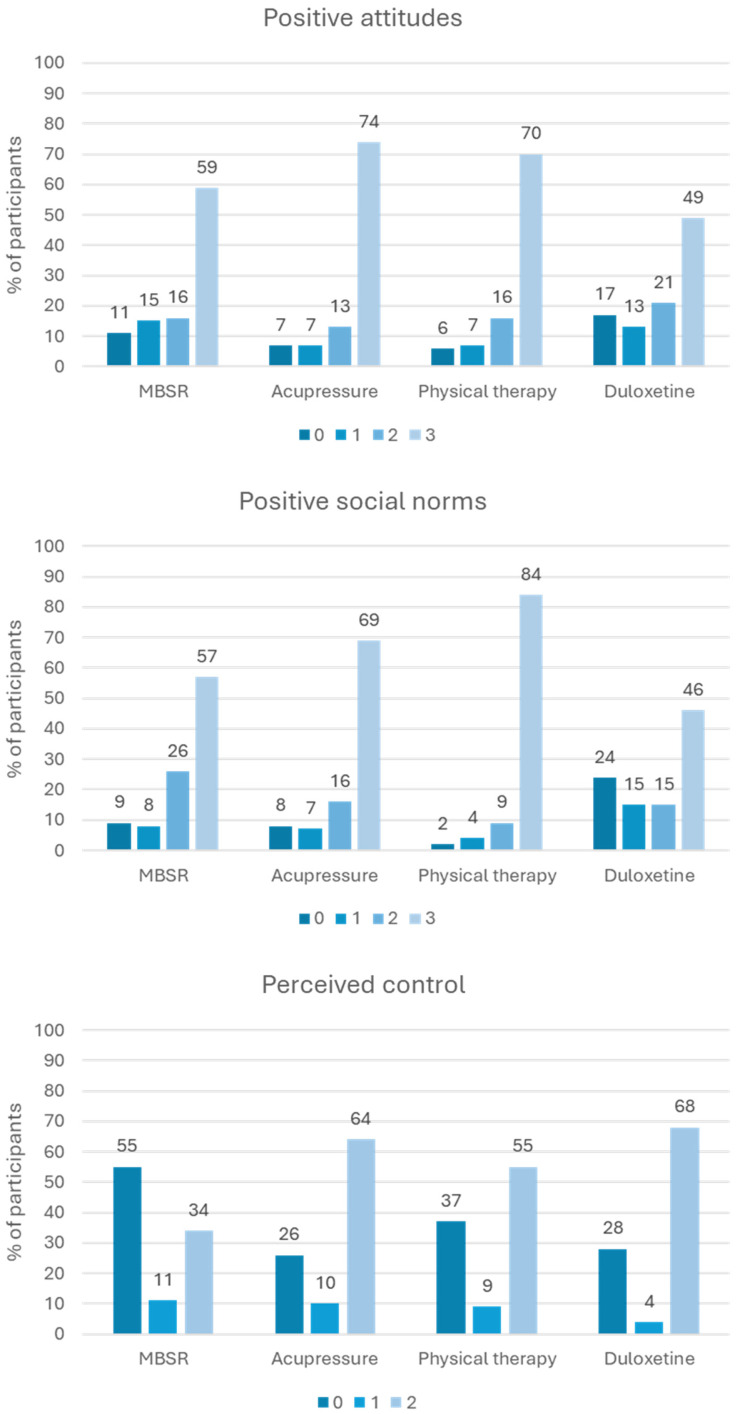
Distribution of theory of planned behavior constructs (positive attitudes, positive social norms, and perceived control) related to each intervention arm for hypothetical chronic low back pain clinical trial. *Note*: Scores indicate the number of items that the participant responds to positively (i.e., for positive attitudes, the number of domains in which the research would be good or pleasant; for social norms, the number of items with which they agree; and for perceived control, the number of ways that the research would be easy to complete). Sample size for responses included: *n* = 361 for MBSR items; *n* = 354 for acupressure items; *n* = 350 for physical therapy items; and *n* = 346 for duloxetine items. The number of items used for perceived control (2 items) was less than for positive attitudes and positive social norms (3 items).

**Figure 2.**
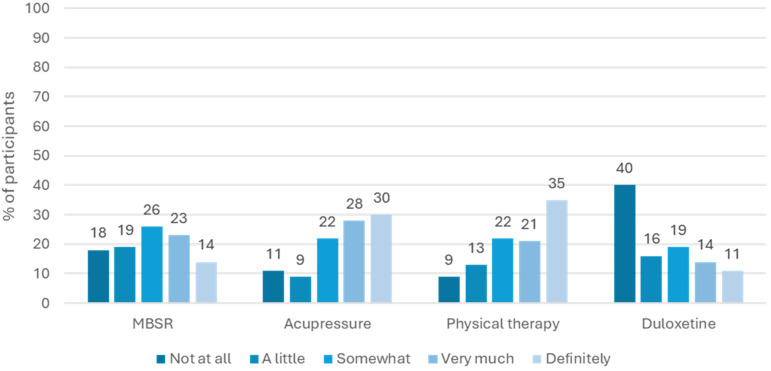
Willingness to participate in each intervention arm for hypothetical chronic low back pain clinical trial. *Note*: Sample size for responses included: *n* = 369 for MBSR; *n* = 356 for acupressure; *n* = 352 for physical therapy; and *n* = 348 for duloxetine.

Response to the TPB constructs varied according to the intervention. See Figure 1. Overall, participants reported positive attitudes and positive perceived social norms for participating in each of the interventions. On the contrary, perceived control displayed a more bivalent structure, with many individuals reporting low perceived control over participating in each of the interventions. Note that the range for perceived control differs from that of positive attitudes and positive social norms because the number of items used differed. As shown in Figure 2, willingness to participate in each intervention was overall positive for acupressure and physical therapy, more evenly dispersed for MBSR, and more negative for duloxetine.

Regression analyses of willingness to participate in a study assigned to MBSR are presented in Table 2. Univariable results suggest that older age was associated with lower willingness to participate. Female gender, higher physical function, higher anxiety symptoms, higher positive attitudes, higher positive social norms, and higher perceived control were all associated with greater willingness to participate. In the multivariable model, higher positive attitudes indicated the greatest odds of increased willingness with 3.25 times higher odds. This was followed by higher perceived control, associated with 2.08 times higher odds of increased willingness, and positive social norms, associated with 1.68 times higher odds of increased willingness.

**Table 2.** Regression analyses predicting willingness to participate in a study assigned to MBSR

		Univariable			Multivariable (*n* = 352)			
		OR	95% CI	*p*	OR	95% CI	*p*	Adjusted *p*
Age		**0.78**	**(0.64, 0.93)**	**0.007**	0.85	(0.67, 1.08)	0.179	0.458
Gender								
	Male (reference)	–	–	–	–	–	–	–
	Female	**2.13**	**(1.40, 3.27)**	**<0.001**	1.64	(1.01, 2.67)	0.045	0.160
	Transgender/non-binary	0.68	(0.17, 2.66)	0.576	0.52	(0.11, 2.35)	0.394	0.768
Race								
	White (reference)	–	–	–	–	–	–	–
	Asian	1.54	(0.87, 2.75)	0.143	1.19	(0.61, 2.33)	0.602	0.840
	Black	1.34	(0.85, 2.12)	0.200	1.01	(0.58, 1.78)	0.965	0.980
	Other	0.96	(0.56, 1.66)	0.886	0.84	(0.44, 1.60)	0.604	0.840
Income over 75,000 USD		0.92	(0.64, 1.33)	0.666	1.14	(0.73, 1.77)	0.563	0.840
Past research participation		0.95	(0.79, 1.13)	0.548	0.90	(0.73, 1.11)	0.327	0.698
Physical function		**1.20**	**(1.00, 1.45)**	**0.050**	1.10	(0.84, 1.45)	0.479	0.829
Anxiety symptoms		**1.39**	**(1.15, 1.68)**	**0.001**	1.19	(0.85, 1.67)	0.311	0.686
Depressive symptoms		1.18	(0.98, 1.42)	0.086	1.11	(0.79, 1.55)	0.553	0.840
Pain intensity		0.96	(0.80, 1.16)	0.679	1.17	(0.89, 1.55)	0.258	0.610
Knowledge of intervention		0.97	(0.81, 1.17)	0.756	1.09	(0.88, 1.35)	0.416	0.783
Positive attitudes		**5.15**	**(3.98, 6.78)**	**<0.001**	**3.25**	**(2.38, 4.49)**	**<0.001**	**<0.001**
Positive social norms		**3.74**	**(2.95, 4.82)**	**<0.001**	**1.68**	**(1.24, 2.29)**	**0.001**	**0.006**
Perceived control		**2.72**	**(2.22, 3.37)**	**<0.001**	**2.08**	**(1.66, 2.61)**	**<0.001**	**<0.001**

*Note*: Boldfaced values are significant at *p* < 0.05. Significance of multivariable findings are based on adjusted *p* values.

Regression analyses of willingness to participate in a study assigned to acupressure are presented in Table 3. Univariable results suggest that individuals identifying as Black were less willing to participate. Female gender, more past research study participation, higher positive attitudes, higher positive social norms, and higher perceived control were associated greater willingness to participate. In the multivariable model, higher positive attitudes indicated the greatest odds of increased willingness with 2.75 times higher odds. This was followed by higher perceived control, associated with 2.07 times higher odds of increased willingness, positive social norms, associated with 1.80 times higher odds of increased willingness, and knowledge of the intervention, associated with 1.37 times higher odds of increased willingness.

**Table 3.** Regression analyses predicting willingness to participate in a study assigned to acupressure

		Univariable			Multivariable (*n* = 346)			
		OR	95% CI	*p*	OR	95% CI	*p*	Adjusted *p*
Age		0.95	(0.79, 1.15)	0.602	1.07	(0.84, 1.35)	0.597	0.840
Gender								
	Male (reference)	–	–	–	–	–	–	–
	Female	**1.77**	**(1.15, 2.74)**	**0.010**	1.53	(0.94, 2.48)	0.086	0.265
	Transgender/non-binary	2.38	(0.65, 9.24)	0.195	0.78	(0.16, 3.70)	0.752	0.870
Race								
	White (reference)	–	–	–	–	–	–	–
	Asian	1.36	(0.73, 2.55)	0.337	2.24	(1.12, 4.56)	0.023	0.092
	Black	**0.54**	**(0.34, 0.86)**	**0.009**	1.22	(0.69, 2.17)	0.495	0.834
	Other	0.70	(0.40, 1.21)	0.196	0.84	(0.44, 1.59)	0.585	0.840
Income over 75,000 USD		1.22	(0.84, 1.78)	0.297	0.91	(0.58, 1.43)	0.690	0.870
Past research participation		**1.34**	**(1.11, 1.64)**	**0.003**	1.26	(1.02, 1.58)	0.037	0.139
Physical function		1.08	(0.89, 1.30)	0.447	1.06	(0.80, 1.40)	0.699	0.870
Anxiety symptoms		0.97	(0.80, 1.17)	0.750	1.02	(0.73, 1.43)	0.895	0.939
Depressive symptoms		0.96	(0.79, 1.16)	0.666	1.15	(0.82, 1.62)	0.430	0.786
Pain intensity		0.97	(0.80, 1.18)	0.781	1.24	(0.93, 1.66)	0.142	0.379
Knowledge of intervention		1.14	(0.93, 1.39)	0.201	**1.37**	**(1.10, 1.69)**	**0.004**	**0.020**
Positive attitudes		**5.11**	**(3.88, 6.88)**	**<0.001**	**2.75**	**(1.96, 3.93)**	**<0.001**	**<0.001**
Positive social norms		**3.57**	**(2.82, 4.58)**	**<0.001**	**1.80**	**(1.31, 2.48)**	**<0.001**	**<0.001**
Perceived control		**3.28**	**(2.63, 4.13)**	**<0.001**	**2.07**	**(1.59, 2.70)**	**<0.001**	**<0.001**

*Note*: Boldfaced values are significant at *p* < 0.05. Significance of multivariable findings are based on adjusted *p* values.

Regression analyses of willingness to participate in a study assigned to physical therapy are presented in Table 4. Univariable results suggest that higher depressive symptoms and higher pain intensity were associated with lower willingness to participate. Female gender, Asian race, more past research study participation, higher physical function, higher knowledge of physical therapy, higher positive attitudes, higher positive social norms, and higher perceived control were associated with greater willingness to participate. In the multivariable model, Asian race (compared to White race) indicated the greatest odds of increased willingness with 2.96 times higher odds. This was followed by higher positive attitudes, associated with 2.27 times higher odds of increased willingness, perceived control, associated with 2.09 times higher odds of increased willingness, and positive social norms, associated with 1.44 times higher odds of increased willingness.

**Table 4.** Regression analyses predicting willingness to participate in a study assigned to physical therapy

		Univariable			Multivariable (n = 342)			
		OR	95% CI	*p*	OR	95% CI	*p*	Adjusted *p*
Age		1.03	(0.85, 1.24)	0.790	1.04	(0.82, 1.31)	0.768	0.870
Gender								
	Male (reference)	–	–	–	–	–	–	–
	Female	**1.76**	**(1.14, 2.74)**	**0.011**	1.31	(0.81, 2.13)	0.267	0.610
	Transgender/non-binary	1.05	(0.30, 3.83)	0.939	1.00	(0.23, 4.57)	0.997	0.997
Race								
	White (reference)	–	–	–	–	–	–	–
	Asian	**2.18**	**(1.17, 4.14)**	**0.015**	**2.96**	**(1.44, 6.26)**	**0.004**	**0.020**
	Black	0.94	(0.59, 1.50)	0.802	1.11	(0.63, 1.97)	0.716	0.870
	Other	0.79	(0.45, 1.39)	0.406	1.19	(0.62, 2.30)	0.599	0.840
Income over 75,000 USD		1.29	(0.89, 1.89)	0.182	1.07	(0.68, 1.69)	0.775	0.870
Past research participation		**1.27**	**(1.06, 1.55)**	**0.014**	1.18	(0.95, 1.47)	0.135	0.376
Physical function		**1.39**	**(1.15, 1.69)**	**0.001**	0.95	(0.71, 1.27)	0.749	0.870
Anxiety symptoms		0.92	(0.76, 1.11)	0.395	1.36	(0.98, 1.89)	0.068	0.229
Depressive symptoms		**0.81**	**(0.67, 0.98)**	**0.031**	0.77	(0.54, 1.08)	0.134	0.376
Pain intensity		**0.75**	**(0.62, 0.91)**	**0.004**	1.03	(0.76, 1.38)	0.866	0.924
Knowledge of intervention		**1.26**	**(1.04, 1.53)**	**0.018**	1.28	(1.04, 1.59)	0.020	0.085
Positive attitudes		**3.50**	**(2.78, 4.44)**	**<0.001**	**2.27**	**(1.70, 3.05)**	**<0.001**	**<0.001**
Positive social norms		**2.71**	**(2.15, 3.48)**	**<0.001**	**1.44**	**(1.10, 1.92)**	**0.009**	**0.041**
Perceived control		**2.84**	**(2.30, 3.53)**	**<0.001**	**2.09**	**(1.64, 2.67)**	**<0.001**	**<0.001**

*Note*: Boldfaced values are significant at *p* < 0.05. Significance of multivariable findings are based on adjusted *p* values.

Regression analyses of willingness to participate in a study assigned to duloxetine are presented in Table 5. Univariable results suggest that higher physical function was associated with lower willingness to participate. Higher pain intensity, higher positive attitudes, higher positive social norms, and higher perceived control were associated with greater willingness to participate. In the multivariable model, higher positive attitudes indicated the greatest odds of increased willingness with 4.42 times higher odds. This was followed by higher social norms, associated with 2.26 times higher odds of increased willingness, and perceived control, associated with 1.99 times higher odds of increased willingness.

**Table 5.** Regression analyses predicting willingness to participate in a study assigned to duloxetine

		Univariable			Multivariable (*n* = 338)			
		OR	95% CI	*p*	OR	95% CI	*p*	Adjusted *p*
Age		1.10	(0.91, 1.34)	0.336	1.06	(0.81, 1.38)	0.677	0.870
Gender								
	Male (reference)	–	–	–	–	–	–	–
	Female	0.86	(0.56, 1.34)	0.514	0.95	(0.56, 1.62)	0.860	0.924
	Transgender/non-binary	0.49	(0.12, 1.75)	0.285	0.71	(0.13, 3.65)	0.687	0.870
Race								
	White (reference)	–	–	–	–	–	–	–
	Asian	0.65	(0.35, 1.19)	0.163	0.72	(0.33, 1.54)	0.396	0.768
	Black	1.16	(0.73, 1.85)	0.532	1.19	(0.63, 2.23)	0.592	0.840
	Other	0.76	(0.42, 1.35)	0.352	0.72	(0.35, 1.46)	0.369	0.762
Income over 75,000 USD		0.73	(0.49, 1.07)	0.104	0.73	(0.44, 1.19)	0.206	0.507
Past research participation		0.99	(0.81, 1.20)	0.918	1.06	(0.83, 1.35)	0.635	0.865
Physical function		**0.71**	**(0.58, 0.87)**	**0.001**	0.89	(0.65, 1.22)	0.468	0.829
Anxiety symptoms		1.10	(0.90, 1.33)	0.363	0.94	(0.65, 1.36)	0.744	0.870
Depressive symptoms		1.17	(0.96, 1.43)	0.113	1.05	(0.73, 1.50)	0.807	0.890
Pain intensity		**1.32**	**(1.09, 1.61)**	**0.005**	1.31	(0.96, 1.80)	0.087	0.265
Knowledge of intervention		0.96	(0.79, 1.17)	0.713	0.99	(0.77, 1.28)	0.952	0.980
Positive attitudes		**7.44**	**(5.31, 10.80)**	**<0.001**	**4.42**	**(2.93, 6.86)**	**<0.001**	**<0.001**
Positive social norms		**4.67**	**(3.60, 6.16)**	**<0.001**	**2.26**	**(1.64, 3.13)**	**<0.001**	**<0.001**
Perceived control		**3.61**	**(2.80, 4.75)**	**<0.001**	**1.99**	**(1.40, 2.86)**	**<0.001**	**<0.001**

*Note*: Boldfaced values are significant at *p* < 0.05. Significance of multivariable findings are based on adjusted *p* values.

## Discussion

Successful clinical trials are necessary for improving interventions for CLBP. The TPB, which has been widely applied to understand health-related behavior in chronic pain patients [16–18], may be a useful model to guide modifiable factors that increase recruitment efforts and potentially downstream effects on retention. Our study suggests that TPB constructs, particularly positive attitudes toward the intervention and perceived control over completing study related tasks, are associated with higher willingness to participate in clinical trials testing specific interventions for CLBP.

Willingness to participate differed across the interventions. Acupressure and physical therapy were both relatively high, MBSR was evenly dispersed, and duloxetine was low. Based on the finding that TPB constructs were robustly linked to willingness to participate, better understanding attitudes, perceived social norms, and perceived control over intervention involvement may help elucidate these differences in willingness.

In general, participants hold positive attitudes about participating in each intervention and believe their physicians and peers would approve of them participating in the interventions. These factors, in turn, were associated with higher willingness to participate. This corresponds to previous research which found that positive attitudes and perceived social norms toward research facilitated participation [13–15]. The unique finding of TPB responses being higher for acupressure and physical therapy may be related to the physical nature of the interventions. Acupressure involves applying pressure to various points on the body and physical therapy includes movement and exercises [25]. While this extends beyond the scope of the current study, it is possible that the physical aspects of the treatments may help participants feel more connected to improving their pain, indicate a preference for interventions that have a physical component, or feel more validating [26]. Notably, positive attitudes and positive social norms were lowest for duloxetine. Participants may feel less positive or be more worried about the norms surrounding taking a medication for their pain. A study by McCracken et al., found that concerns about addiction, adverse scrutiny, side effects, tolerance, and mistrust of the prescribing doctor were all negatively associated with adherence to pain medications [27]. According to the TPB, beliefs predict attitudes, which then are more proximal to the evaluation of willingness. Thus, although attitudes may be most fruitful for understanding willingness, future research may benefit from better understanding the beliefs related to more positive attitudes and perceived social norms.

Interestingly, we found more variation in responses related to perceived control. Participants understandably perceived the lowest amount of control over completing time-intensive interventions such as MBSR or interventions that require clinic visits such as physical therapy. Alternatively, interventions that are self-administered and require little time, herein including acupressure and duloxetine, were generally perceived as controllable. This corresponds to the finding that time limitations are a potent barrier to research participation [28–29].

### Research implications and future directions

The current findings suggest that efforts to improve TPB constructs, possibly through study messaging, may be fruitful for improving willingness to participate in clinical trials for CLBP [30]. It is unclear if willingness indeed translates to participation. Future longitudinal studies should investigate the correspondence between willingness and actual participation. Notably, the TPB suggests that low perceived control attenuates the association between willingness or intentions and actual behavior. This is highlighted by the finding that barriers to research participation are important to understanding recruitment and retention. Thus, concerted efforts should be made to both improve attitudes and perceived norms, as well as reduce barriers to participation. Researchers may find it beneficial to implement TPB constructs early in the study design process by probing attitudes, social norms, and perceived control considerations with patient advisory boards. Such information can not only be used to inform study recruitment, but also to develop a study that is perceived positively and lessens the burden for participants. A TPB-informed trial design may reduce attrition rates, save money, and increase statistical power.

Increasing positive attitudes may also have an impact on treatment effects in a clinical trial. If a participant feels more positively about their participation in a trial or the treatment they are receiving, that positive attitude alone could result in therapeutic benefit. Previous research findings have shown that pessimists are more likely to have a negative impact than optimists in certain treatment groups [31]. Past research has also suggested that TPB factors may increase susceptibility to hypnotherapy by enhancing the participant’s belief that they are hypnotizable [32]. Treatment expectancies are well-acknowledged and assessed in clinical trials and TPB constructs may further augment our understanding of these effects. Researchers should also be cognizant about what recruitment and retention strategies (such as targeting TPB-related beliefs and attitudes) may impact expectancies.

### Limitations

There are numerous limitations to the present study. The participants who completed this survey were a convenience sample of Michigan Medicine patients; therefore, the findings may not be generalizable to other individuals with CLBP. The survey was also only accessible to participants with internet access. Additionally, the majority of the sample were White women, which also limits generalizability. The number of eligible participants who did not complete the survey was not tracked, making it difficult to measure the representativeness of the sample. The predominance of positive responses may reflect the limited way in which the TPB constructs were assessed, which were simply checklist items. We included four specific interventions that were relevant to current recruitment efforts; it is unclear how TPB constructs may translate to other CLBP interventions. The study was cross-sectional; it was not possible to evaluate how willingness corresponds to actual participation or to assess causality. Future research would benefit from longitudinal tracking of research participation through recruitment and retention. Finally, the order in which the treatment specific sections of the questionnaire were given was not randomized, which could have resulted in respondent fatigue toward the end of the survey.

## Conclusion

Overall, we found that constructs of the TPB are strong predictors of willingness to participate in CLBP clinical trials. Future research should extend the current findings to evaluate whether using these TPB constructs to tailor study materials could improve clinical trial recruitment, retention, and treatment adherence. These applications could improve clinical trials leading to more effective research and improvements in the field of CLBP treatment.

## Supporting information

10.1017/cts.2025.10209.sm001 Steeby et al. supplementary material Steeby et al. supplementary material

## Supplementary material

The supplementary material for this article can be found at https://doi.org/10.1017/cts.2025.10209.
