# Supplementary material for: Theory of planned behavior constructs are associated with willingness to engage in clinical trial interventions for chronic low back pain: A cross-sectional survey study
Source: J Clin Transl Sci. 2025 Dec 19;9(1):e283. doi: 10.1017/cts.2025.10209 (PMC12780809; doi:10.1017/cts.2025.10209)
Supplement: Steeby et al. supplementary material [file S2059866125102094sup001.docx]

**Supplemental Table 1:**

Additional information was provided in the survey about the intervention and what study tasks would be required. Participants were presented with the information listed below prior to completing each respective section of the survey.

**Mindfulness-based stress reduction**

Here’s more information:

Mindfulness-based stress reduction (MBSR) is a structured 8-week program that trains people to use mindfulness practices. It combines meditation, body awareness, and changing thoughts, feelings, and behavior to improve pain, functioning and well-being.

The program is led by a therapist. It includes 8 weekly 1-hour group sessions, 1 weekend retreat, and up to one hour of daily homework. The 1-hour sessions and retreat can all be completed over a video call like Zoom. The daily homework is completed by the participant on their own time.

**Acupressure**

Here’s more information:

Acupressure includes the use of a device to apply pressure to specific points of the body with the goal of relieving pain.

The acupressure program involves 30 minutes of self-administered acupressure each day using an app to guide you. In addition you will take 3-5 minutes to record a pain score and time of acupressure completion in a daily log.

**Physical Therapy**

Here’s more information:

Physical therapy is a treatment that uses exercises and equipment to regain or improve movement and/or function.

The physical therapy program involves ten one-hour sessions: 2 sessions per week for the first 2 weeks and then 1 session per week for the following 6 weeks. The regular completion of a variety of at-home stretching/walking/assigned exercises will also be expected following the formal sessions.

**Duloxetine**

Here’s more information:

Duloxetine is an FDA-approved prescription medication that can be taken daily to help manage chronic pain.

The duloxetine treatment involves taking up to two pills daily at similar approximate times each day for about 8 weeks.
